# Laparoscopic-Assisted Ovary-Sparing Surgery for Ovarian Torsion in Children and Adolescents: A Single-Center 6.4-Year Retrospective Analysis

**DOI:** 10.3390/jcm15208007

**Published:** 2026-10-15

**Authors:** Ming Liu, Jinwen Meng, Xiongwei Wu, Jiabin Cai, Min He, Jinhu Wang

**Affiliations:** 1 Department of Surgical Oncology, Children’s Hospital, Zhejiang University School of Medicine, Hangzhou 310009, China; ming_liu@zju.edu.cn (M.L.);; 2 National Clinical Research Center for Children and Adolescents’ Health and Diseases, Hangzhou 310009, China

**Keywords:** one-stage ovarian tumor enucleation, ovarian tumor, torsion of pedicle, children, surgery, laparoscopy

## Abstract

**Objectives**: To explore the surgical experience and clinical value of laparoscopic exploration with one-stage ovarian tumor enucleation and oophoroplasty for ovarian tumor pedicle torsion in children and adolescents. **Methods**: A retrospective analysis was conducted on the clinical data of 105 cases of ovarian torsion managed at the Department of Oncological Surgery, Children’s Hospital, Zhejiang University School of Medicine from 1 January 2019 to 1 June 2025. All patients underwent emergency laparoscopic exploration with one-stage ovarian tumor enucleation and oophoroplasty. Postoperative ovarian Doppler ultrasound follow-up was performed at 1–3 months, 3–6 months, 6–12 months, and over 12 months, comparing the ovarian volume of the affected side with that of the contralateral ovary. **Results**: This study included 105 cases of ovarian tumor pedicle torsion. The patients ranged in age from 11 months to 14 years, with a median age of 8 years. The surgery was successfully completed in all 105 cases. Ovarian survival was achieved in 103 cases (98.09%), postoperative ovarian necrosis occurred in 1 case (0.95%), and the affected ovary and fallopian tube were removed due to malignant tumor in 1 case (0.95%); the overall complication rate was 13.3% (14/105). Comparison of ovarian volume between the affected side and the contralateral ovary at each postoperative follow-up time point: at 1–3 months postoperatively, the volume was 3.28 (1.33, 7.71) cm^3^ vs. 3.43 (1.38, 5.92) cm^3^ (*p* = 0.301); at 3–6 months postoperatively, the volume was 1.28 (0.76, 6.09) cm^3^ vs. 2.67 (1.23, 6.24) cm^3^ (*p* = 0.169); at 6–12 months postoperatively, the volume was 3.37 (2.04, 6.66) cm^3^ vs. 4.37 (2.02, 6.28) cm^3^ (*p* = 0.092); at more than 12 months postoperatively, the volume was 4.07 (1.10, 5.04) cm^3^ vs. 5.60 (1.02, 9.85) cm^3^ (*p* = 0.090). At all follow-up time points, the comparison of ovarian volume between the affected side and the contralateral ovary showed no statistically significant difference (all *p* > 0.05). **Conclusions**: Emergency laparoscopic exploration with concurrent tumor enucleation and oophoroplasty for ovarian tumor pedicle torsion in children and adolescents is safe and feasible. It reduces the need for a second surgery and effectively preserves ovarian tissue on the affected side.

## 1. Introduction

Ovarian tumor pedicle torsion is one of the common acute abdominal conditions in pediatric surgery. To reduce the long-term risk of infertility and minimize negative social and emotional impacts in pediatric and adolescent patients, ovary-sparing surgery (OSS) can prevent premature ovarian failure, osteoporosis, cardiovascular disease, and other long-term risks. Preserving as much ovarian tissue as possible during surgery has become a clinical consensus [1,2,3]. Currently, laparoscopic exploration and detorsion of the twisted ovary and tumor are recognized as the preferred surgical approach.

However, current domestic and international studies have not provided clear recommendations on whether to perform one-stage ovarian tumor enucleation after detorsion [4]. After ovarian torsion, impaired venous return causes congestion and edema of the ovarian tissue. Intraoperatively, the ovary often appears purplish-black and does not completely resolve within a short period. Current consensus on managing ovarian torsion in children and adolescents suggests that performing tumor enucleation åat the initial surgery solely because the ovary appears swollen and dark may further damage the already ischemic and edematous ovarian tissue. Therefore, it is recommended to perform an ultrasound examination 6–8 weeks after detorsion, and once the blood supply to the affected ovary is restored and the edema has subsided, elective tumor enucleation can be performed [1,3].

However, this recommendation is based largely on pathophysiological reasoning and retrospective observations. To date, prospective comparative evidence directly assessing whether one-stage tumor enucleation is as safe and effective as delayed, staged enucleation in children and adolescents remains scarce. The available evidence is heterogeneous, with findings that are not yet consistent; further research is therefore needed. This study retrospectively analyzed the case data of children who underwent emergency detorsion combined with one-stage tumor enucleation for ovarian tumor torsion in our hospital, aiming to explore the safety and feasibility of one-stage surgery and to provide a reference for the clinical management of ovarian tumor pedicle torsion in children and adolescents.

## 2. Materials and Methods

### 2.1. Ethics Approval Statement

Institutional Review Board Statement: The study was conducted in accordance with the Declaration of Helsinki and approved by the Ethics Committee of Children’s Hospital, Zhejiang University, School of Medicine (protocol code: 2026-IRB-0211-P-01 and date of approval: 2 June 2026). Given the retrospective design and the use of de-identified patient data, the requirement for informed consent was waived by the Institutional Review Board.

### 2.2. Clinical Data

**Figure 1:**
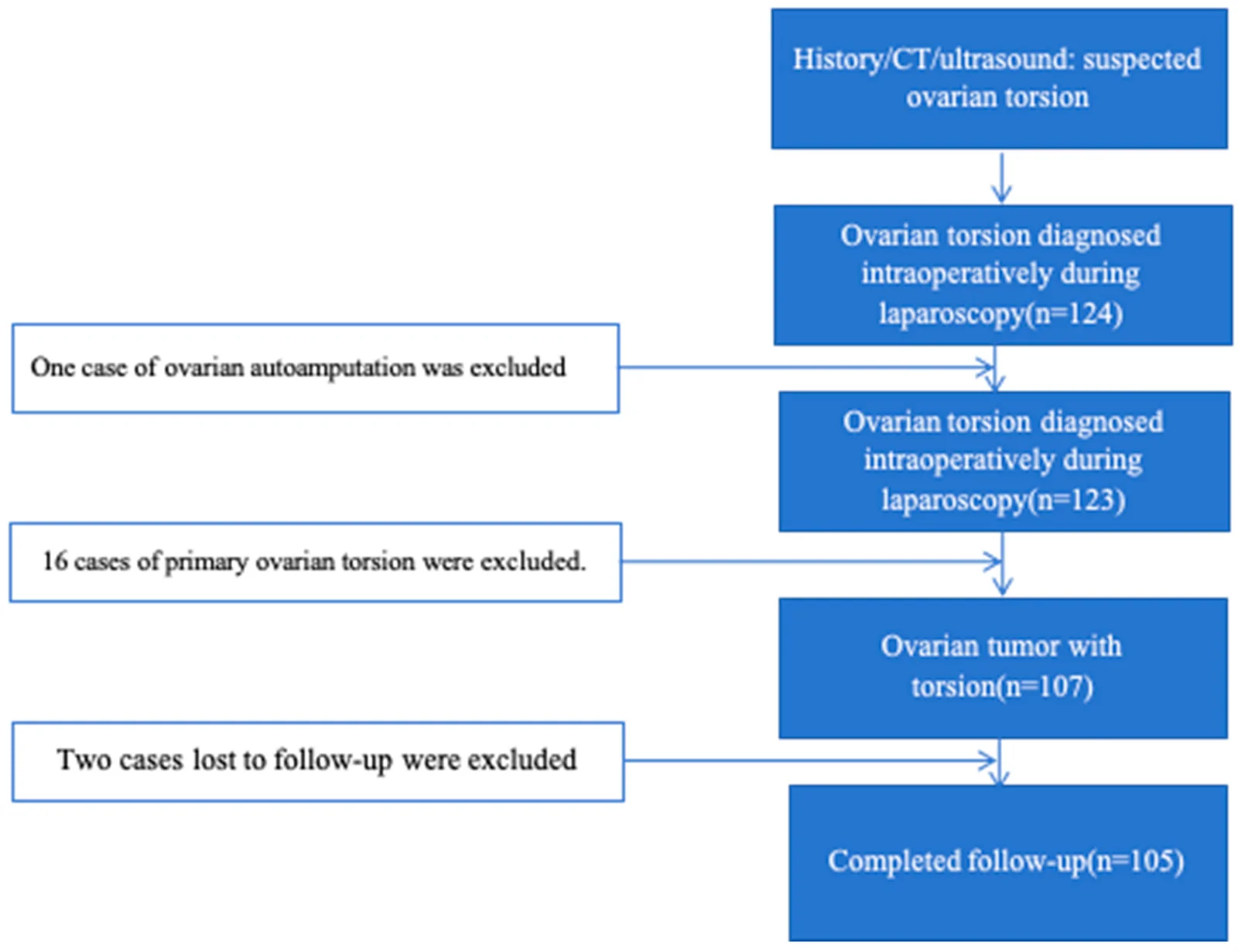
Ovarian tumor with pedicle torsion: case selection flowchart for one-stage ovarian tumor enucleation.

The case data of patients with ovarian tumor torsion who underwent surgical treatment in the Department of Oncological Surgery, Children’s Hospital, Zhejiang University School of Medicine from 1 January 2019 to 1 June 2025 were retrospectively analyzed. Follow-up was completed on July 1, 2026. Patient selection and exclusion criteria are summarized in a flow diagram (Figure 1). We initially identified 124 potentially eligible cases. Cases were excluded for the following reasons: one case of ovarian autoamputation (*n* = 1), cases of primary ovarian torsion (*n* = 16), and patients with incomplete clinical data. (*n* = 2). Finally, 105 patients were included in the analysis.

We excluded primary ovarian torsion cases because their surgical procedure differs (no tumor enucleation is required); including them would confound the analysis of tumor-associated torsion.

### 2.3. Methods

This study enrolled a total of 105 eligible pediatric cases. All patients underwent emergency laparoscopic exploration after admission, during which torsion of an ovarian tumor was confirmed intraoperatively, and one-stage ovarian tumor enucleation with oophoroplasty was performed. Postoperative Doppler ultrasonographic follow-up, performed using a GE HealthCare ultrasound system (GE HealthCare, Wuxi, China), demonstrated blood perfusion in the ovarian tissue, and the presence of viable follicles was considered indicative of ovarian survival. Follow-up ultrasound examinations were carried out at 1–3 months, 3–6 months, 6–12 months, and more than 12 months after surgery, and ovarian volume on the affected side was compared with that of the contralateral healthy ovary. Meanwhile, ovarian blood supply was assessed by transabdominal Doppler ultrasonography, and the ovarian survival rate following one-stage ovarian-sparing surgery as well as the incidence of postoperative complications were statistically analyzed. Ovarian volume was calculated using the standard prolate ellipsoid formula: Volume = Length × Width × Height × 0.523. Because this was a retrospective review with long-term follow-up, the postoperative ultrasound examinations were performed by different experienced sonographers on duty.

The grading of ovarian torsion was jointly determined by two surgeons. When discrepancies occurred, the final grade was adjudicated by a senior surgeon (J.W) according to standardized intraoperative photographic records to ensure reliability. The grading of ovarian torsion is as follows. In cases of discrepancy, consensus was reached through intraoperative discussion.

Grade 1: The ovary exhibits slight discoloration without marked enlargement. Following detorsion, the color returns to normal.

Grade 2: The ovary appears dark red to brown, and edema of the ovarian tissue is present. After detorsion, the color becomes redder, but petechiae are observed on the surface.

Grade 3: The ovary is brown to black in appearance, and the blue–black sign is noted. Tissue edema is evident. Following detorsion, a slight improvement in color is observed.

Grade 4: The ovary appears completely black or pale, with significant enlargement. After detorsion, no improvement in the gross appearance or color of the ovary is observed.

### 2.4. Statistical Methods

Statistical analysis was performed using SPSS 27.0 software. Categorical variables were expressed as frequencies and percentages [*n* (%)]. Data on patient age, symptom duration, preoperative and postoperative ovarian size, and degree of ovarian torsion exhibited a skewed distribution and were presented as median and interquartile range [Median (IQR)]. Variables following a normal distribution were expressed as mean ± standard deviation (mean ± SD). Ovarian volumes of the affected and unaffected ovaries at each postoperative follow-up time point showed a skewed distribution, and comparisons between the two sides were conducted using the Wilcoxon signed-rank test. A *p* value of <0.05 was considered statistically significant. This study was approved by the Ethics Committee of the Children’s Hospital, Zhejiang University School of Medicine.

## 3. Results

### 3.1. Baseline Characteristics

A total of 105 cases of ovarian tumor pedicle torsion were included in this group. The age distribution was skewed, with a median (IQR) of 8 (5, 11) years. Of these, 22 patients (20.95%) had reached menarche, and 83 (79.05%) had not. Torsion occurred on the right side in 60 cases (57.14%) and on the left side in 45 cases (42.86%). Initial symptoms: abdominal pain alone in 36 cases (34.29%), abdominal pain with vomiting in 65 cases (61.90%), and abdominal pain with fever in 4 cases (3.81%). The time from symptom onset to surgery showed a skewed distribution, Median (IQR) is 29.0 (16.0, 48.0) hours.

### 3.2. Intraoperative Findings

#### 3.2.1. Surgical Procedure

A 10 mm incision was made at the left periumbilical margin, a Veress needle was inserted, CO_2_ was insufflated to establish a pneumoperitoneum, and intra-abdominal pressure was maintained at 8–12 mmHg. Through the same incision, we inserted a 10 mm trocar and laparoscope and explored the abdominal cavity to confirm torsion of the affected ovary. We made small incisions of approximately 5 mm in the left lower abdomen and right abdomen and inserted 5 mm trocars along with operative instruments including grasping forceps or dissecting forceps. We observed the color and degree of torsion of the ovary, and then detorsed the twisted ovary and fallopian tube by gently rotating them in the opposite direction. Using electrocautery (cutting mode, low power), we incised the ovarian cortex to expose the internal tumor, then completely enucleated the tumor while preserving as much normal ovarian tissue as possible. The ovarian wound was closed with continuous 3-0 or 4-0 absorbable barbed suture for hemostasis. The tumor was placed in a retrieval bag, and after appropriate enlargement of the incision, it was completely removed. We re-established pneumoperitoneum, irrigated the pelvic cavity, and after confirming no active bleeding, evacuated the pneumoperitoneum, withdrew the instruments, and closed the abdomen in layers.

**Figure 2:**
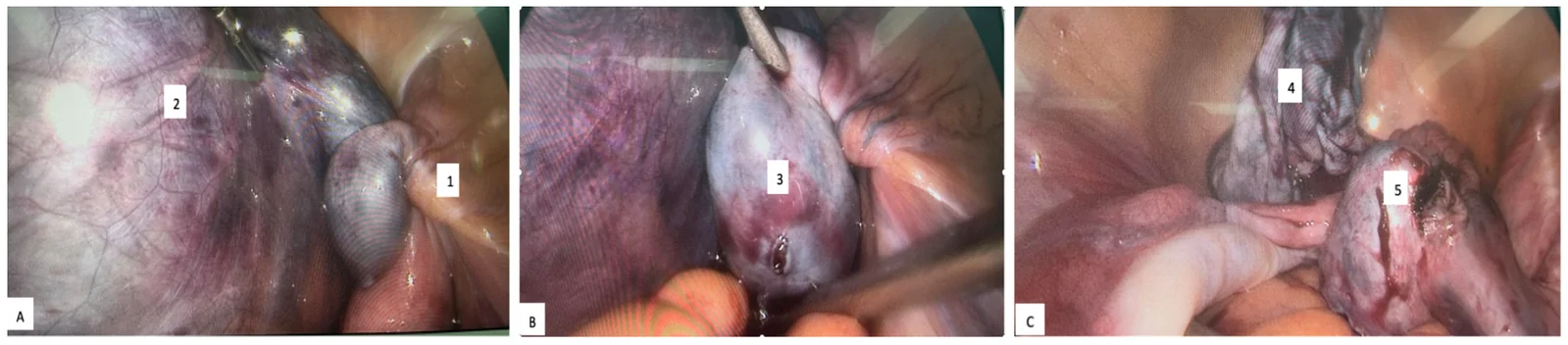
(**A**) Detorsion and assessment of the twisted adnexa. (**B**) Enucleation of the ovarian tumor. (**C**) Oophoroplasty and hemostasis. 1: Twisted pedicle of the adnexa; 2: Twisted ovarian tumor; 3: Dark red, edematous ovarian parenchyma; 4: Enucleated ovarian tumor; 5: Preserved ovarian parenchyma after tumor enucleation. The figure shows a typical case, not a median case.

The intraoperative findings of the ovary are shown in Figure 2.

All surgeries were performed by the same surgical team using standard laparoscopic equipment (Karl Storz, Tuttlingen, Germany).

A twisted right adnexal mass was identified. The pedicle (arrow 1) exhibited a 1080° counterclockwise torsion with a dark, congested appearance. The ovarian tumor (arrow 2) was engorged, and the adjacent ovarian parenchyma (arrow 3) appeared edematous and dark red, suggesting venous stasis rather than irreversible arterial ischemia.

After detorsion and incision of the ovarian cortex, the tumor was carefully dissected from the surrounding parenchyma. The enucleated tumor (arrow 4) measured approximately 3.5 × 3 × 3 cm, had an intact capsule, and contained adipose tissue, hair, and cartilage-like components, consistent with a mature teratoma.

Following complete tumor enucleation, the preserved ovarian parenchyma (arrow 5) was closed with continuous 4-0 absorbable barbed sutures. Adequate hemostasis was achieved without excessive electrocautery, allowing maximal preservation of healthy ovarian tissue. The fallopian tube (FT) appeared viable after detorsion.

#### 3.2.2. Surgical Details 

The operative time was (91.94 ± 28.21) min. The direction of torsion was identified intraoperatively, with clockwise torsion observed in 67 cases (63.81%) and counterclockwise torsion in 38 cases (36.19%). The degree of torsion showed a skewed distribution, with a median (IQR) of 540° (360–900°); torsion ≥ 360° was noted in 99 cases (94.29%). Based on intraoperative findings, ovarian torsion was graded as follows: grade 1 in 19 cases (18.10%), grade 2 in 14 cases (13.33%), grade 3 in 28 cases (26.67%), and grade 4 in 44 cases (41.90%). See Table 1.

### 3.3. Postoperative Results

Postoperatively, seven cases (6.67%) developed fever accompanied by elevated infection markers; their symptoms resolved after anti-infective therapy with second-generation cephalosporins. Five cases (4.76%) had asymptomatic ovarian fluid collection, and two cases (1.90%) developed wound infections. Postoperative pathology revealed: mature cystic-solid teratoma in 81 cases (77.14%), corpus luteum cyst with hemorrhage in 9 cases (8.57%), follicular cyst in 5 cases (4.76%), mucinous cystadenoma in 3 cases (2.86%), serous cystadenoma in 2 cases (1.90%), I grade immature teratoma in 3 cases (2.86%), dysgerminoma in 1 case (0.95%), and sex cord-stromal tumor in 1 case (0.95%). Intraoperative frozen section was not routinely performed. The patient with dysgerminoma subsequently underwent surgical resection of the affected ovary and omentum. See Table 1.

### 3.4. Postoperative Follow-Up Results

At the 1–3, 3–6, 6–12, and >12-month follow-up windows, available ultrasound measurements were recorded in 103 (98.09%), 96 (91.42%), 87 (82.86%), and 90 (85.71%) of the 105 included cases, respectively. The longest follow-up was 47 months. The results showed that the ovary survived in 103 cases (98.09%), ovarian necrosis occurred in 1 case (0.95%), and the affected ovary and fallopian tube were removed due to malignant tumor in 1 case (0.95%). The results showed that the ovary survived in 103 cases (98.09%), ovarian necrosis occurred in 1 case (0.95%), and the affected ovary and fallopian tube were removed due to malignant tumor in 1 case (0.95%). Comparison of ovarian volume between the affected side and the contralateral ovary at each postoperative follow-up time point: at 1–3 months postoperatively, the volume was 3.28 (1.33, 7.71) cm^3^ vs. 3.43 (1.38, 5.92) cm^3^ (*p* = 0.301); at 3–6 months postoperatively, the volume was 1.28 (0.76, 6.09) cm^3^ vs. 2.67 (1.23, 6.24) cm^3^ (*p* = 0.169); at 6–12 months postoperatively, the volume was 3.37 (2.04, 6.66) cm^3^ vs. 4.37 (2.02, 6.28) cm^3^ (*p* = 0.092); and at more than 12 months for cases followed for >12 months postoperatively (*n* = 90), the median (IQR) follow-up duration was 21.3 (14.5–26.0) months. The longest follow-up was 47 months. The affected ovary tended to be smaller than the contralateral ovary, with a median (IQR) volume of 4.07 (1.10–5.04) cm^3^ versus 5.60 (1.02–9.85) cm^3^. with no statistically significant difference (all *p* > 0.05). See Table 2.

## 4. Discussion

Surgery is the gold standard for diagnosing ovarian tumor pedicle torsion, and the current consensus both domestically and internationally is that ovary-sparing surgery (OSS) is the standard treatment for ovarian tumor torsion [1,4]. However, with a deeper understanding of the pathophysiological mechanisms of ovarian torsion and the popularization of laparoscopic techniques, increasing evidence indicates that the intraoperative appearance of the ovary does not accurately reflect its actual viability; even if it appears ischemic, edematous, or even bluish-purple, there is still a high probability of functional recovery after detorsion [1,2,3,4]. The findings of this study further support this view, indicating that for children and adolescents with ovarian tumor torsion, laparoscopic exploration with one-stage tumor enucleation and oophoroplasty is safe and feasible, does not exacerbate ovarian injury, and achieves excellent ovarian survival.

With the widespread adoption of laparoscopic techniques, laparoscopic exploration has become the preferred surgical procedure for ovarian tumor torsion. Under laparoscopic visualization, tumor size, vascularity, degree of edema, and extent of torsion can be clearly assessed [5]. In this study, it is suggested that early laparoscopic exploration should be performed for patients with a suspected diagnosis of ovarian torsion. Once ovarian torsion is confirmed intraoperatively, detorsion should be carried out immediately to restore ovarian blood supply and reduce venous congestion. No thromboembolic events were observed in this study, a finding consistent with multiple previous studies indicating that the clinical incidence of thromboembolic complications following ovarian detorsion is extremely low [6,7].

Based on our surgical experience, enucleating an ovarian tumor from a congested ovary under intraoperative conditions can easily cause tumor rupture and spillage. However, ovarian tumors in children and adolescents are predominantly benign, with mature teratoma being the most common pathological type. For benign ovarian tumors, ovary-sparing tumor enucleation is the preferred approach, requiring complete tumor excision while preserving normal ovarian tissue. Intraoperative tumor rupture and spillage do not increase the risk of recurrence [8]. In our cohort, spillage occurred in 5 patients (4.76%), complete enucleation of the tumor capsule was achieved in all cases, and no recurrence was observed during follow-up. Studies have shown that there is no statistically significant difference in postoperative complications, readmission rates, and recurrence rates between ovary-sparing surgery and adnexectomy [9,10]. The data from this study further confirm that no serious complications of Clavien–Dindo grade II or higher occurred in this group of patients postoperatively.

All 105 patients in this group underwent emergency surgical exploration. Intraoperative frozen section examination was not performed, and the final diagnosis of all tumors was based on postoperative paraffin-embedded histopathology. Among them, there were 5 cases of malignant tumors (4.76%), including 1 case of dysgerminoma, 1 case of sex cord-stromal tumor, and 3 cases of grade I immature teratoma. For the patient with dysgerminoma, a second laparoscopic exploration was performed to remove the affected ovary and fallopian tube, supplemented with chemotherapy [11]. The patient is currently growing and developing well, with no recurrence after 5 years of follow-up. During follow-up of the patient with sex cord-stromal tumor, a mass was found in the contralateral ovary; after reoperation and cystectomy, the pathology remained sex cord-stromal tumor, and at the 5th year of follow-up, no signs of recurrence were observed on either side. The 3 patients with grade I immature teratoma did not receive chemotherapy postoperatively and were followed up regularly as outpatients for 2, 2, and 6 years, respectively, with no evidence of recurrence. These data indicate that even without intraoperative frozen pathology, postoperative management based on paraffin results and the ovary-sparing surgical strategy are safe in children and adolescent patients.

Some scholars believe that after detorsion for ovarian torsion, the ovary becomes black, making accurate assessment of the blood supply impossible, and the ovarian tissue is edematous and fragile; primary surgery may cause further ovarian damage and impair ovarian function. Thus, they recommend performing a second-stage tumor enucleation 6–8 weeks after detorsion, once the ovarian blood supply has recovered [4]. A prospective study from China including 71 children with ovarian torsion reported an overall ovarian preservation rate of 98.60% after conservative surgery, with no thromboembolic events or recurrent torsion. In the subgroup of 34 patients with ovarian tumors treated by staged surgery, 33 had benign tumors and all retained their ovaries, whereas one patient with a granulosa cell tumor ultimately required adnexectomy, giving a preservation rate of 97.1% (33/34) [12]. In our one-stage cohort, the ovarian preservation rate was 98.10%, which is comparable to that reported for staged surgery. These findings suggest that one-stage tumor enucleation with ovarian preservation may offer safety similar to that of a staged approach.

Although the differences in ovarian volume between the affected and contralateral ovaries did not reach statistical significance at any time point, the *p* values at 6–12 months (*p* = 0.092) and >12 months (*p* = 0.090) were borderline, suggesting a possible trend toward volume reduction on the affected side. As shown in Table 2, the affected ovary was indeed smaller at three of the four time points. The median difference (95% CI) progressed from −0.15 (−0.98, 0.67) at 1–3 months to −1.53 (−3.30, 0.24) at >12 months, indicating a widening trend in this difference. The wide confidence intervals, which crossed zero, precluded demonstration of equivalence, and this widening trend suggests a potential long-term effect of volume reduction in the affected ovary, a finding consistent with a previous study [13]. Nevertheless, the ovarian survival rate in this cohort was 98.10%, which is satisfactory. The primary clinical goal of ovarian preservation was achieved in the vast majority of patients. Future studies with larger sample sizes and equivalence-based designs are warranted to confirm whether such volume reduction represents impaired ovarian function.

Studies have demonstrated that postoperative ovarian size and follicular activity are not significantly associated with the degree of intraoperative ovarian ischemia [13], and that anti-Müllerian hormone (AMH), a reliable indicator of ovarian reserve, is positively correlated with ovarian volume [14,15]. The retrospective design of this study precluded the acquisition of AMH measurements, as recalling participants for additional blood sampling was not practically feasible. Consequently, AMH will be prospectively incorporated into this cohort in future phases, enabling a more comprehensive and objective appraisal of ovarian reserve. In the present study, postoperative Doppler ultrasound follow-up was performed in this study to compare ovarian volume of the affected and contralateral ovaries. The results showed that at each follow-up time point, there was no statistically significant difference in ovarian volume between the two sides (all *p* > 0.05), suggesting that one-stage ovarian tumor enucleation and oophoroplasty is safe and feasible in terms of ovarian survival for children with ovarian tumor pedicle torsion. Notably, while all comparisons failed to reach statistical significance, the *p* values showed a decreasing trend over time (0.301, 0.169, 0.092, 0.090), suggesting a possible trend toward smaller affected-side volume at longer follow-up. This trend, however, did not achieve statistical significance, which may reflect limited statistical power due to decreasing sample sizes at later time points. The observed pattern warrants further investigation in larger prospective cohorts with adequate power to detect clinically meaningful differences.

We suggest that the gross appearance of the ovary after detorsion and the subjective intraoperative assessment of ischemia severity are not reliable indicators for deciding whether to delay surgery. After detorsion, a dark purple or black ovary (grade 3–4) is predominantly attributable to venous stasis rather than arterial ischemia; even when the torsion exceeds two complete twists, the ovary can still retain some arterial blood supply and remain viable after detorsion [16]. A retrospective analysis of our cases revealed that, even when the ovary appeared black, blue–black, or purple-black after intraoperative detorsion, one-stage ovarian tumor enucleation could be performed if the torsion was caused by an ovarian tumor. During enucleation, fresh bleeding from the ovarian wound was observed, indicating that arterial blood supply to the ovarian tissue was preserved. Excessive electrocoagulation for hemostasis is not recommended, as it may damage the ovarian tissue [17]; instead, continuous suturing of the ovarian wound with a 3-0 or 4-0 barbed suture is advised to achieve hemostasis, and this approach can preserve ovarian tissue.

In conclusion, laparoscopic exploration, detorsion of the twisted ovary, and one-stage ovarian tumor enucleation with oophoroplasty was feasible and associated with a high ovarian survival rate.

Study limitations: 1. This is a single-arm retrospective study lacking a concurrent control group that underwent staged surgery or adnexectomy, which limits our ability to directly compare outcomes between different surgical strategies. 2. As the postoperative ultrasound assessments were conducted as part of routine follow-up care, the sonographers were not blinded to the affected side or clinical presentation. This limitation may have contributed to inter-observer variability and observer bias. 3. Although ovarian volume correlates with ovarian reserve, it is not a direct measure of endocrine function or fertility potential. Future long-term studies incorporating AMH levels and fertility outcomes are needed to fully validate the efficacy of this approach. Future prospective studies incorporating anti-Müllerian hormone (AMH) are planned to further assess ovarian reserve and long-term functional outcomes.

## Figures and Tables

**Table 1:** Baseline characteristics and intraoperative findings of the 105 patients.

Characteristic	N (%)/M (IQR)/Mean ± SD
**Age**	8 (5, 11)
**Menarche**	
Yes	22 (20.95%)
No	83 (79.05%)
**Symptom duration**	29 (16, 48)
**Abdominal pain**	36 (34.29%)
**Abdominal pain and vomiting**	65 (61.90%)
**Abdominal pain and fever**	4 (3.81%)
**Torsion side**	
Right	60 (57.14%)
Left	45 (42.86%)
**Preoperative ultrasound findings**	
Affected Side Volume	69.93 (6.02, 167.34)
Operative time	91.94 ± 28.21
Grading of ovarian torsion	
Grade 1	19 (18.10%)
Grade 2	14 (13.33%)
Grade 3	28 (26.67%)
Grade 4	44 (41.90%)
**Torsion direction**	
Clockwise	67 (63.81%)
Counterclockwise	38 (36.19%)
Degree of torsion, median	540 (360, 900)
Intraoperative rupture or spillage	5 (4.76%)
**Postoperative complications**	14 (13.3%)
Wound infection	2 (1.90%)
Fever	7 (6.67%)
Asymptomatic ovarian fluid collection	5 (4.76%)
**Postoperative pathology**	
Mature cystic teratoma	81 (77.14%)
Hemorrhagic corpus luteum cyst	9 (8.57%)
Follicular cyst	5 (4.76%)
Mucinous cystadenoma	3 (2.86%)
Serous cystadenoma	2 (1.90%)
Grade I immature teratoma	3 (2.86%)
Sex cord-stromal tumor	1 (0.95%)
Dysgerminoma	1 (0.95%)

The grading of ovarian torsion is as follows: Grade 1: The ovary exhibits slight discoloration without marked enlargement. Following detorsion, the color returns to normal. Grade 2: The ovary appears dark red to brown, and edema of the ovarian tissue is present. After detorsion, the color becomes redder, but petechiae are observed on the surface. Grade 3: The ovary is brown to black in appearance, and the blue–black sign is noted. Tissue edema is evident. Following detorsion, a slight improvement in color is observed. Grade 4: The ovary appears completely black or pale, with significant enlargement. After detorsion, no improvement in the gross appearance or color of the ovary is observed.

**Table 2:** Follow-up rates and comparison of ovarian volumes between the affected and contralateral sides.

Follow Up Time Window	Cases with Available Data, *n* (%)	Affected Side Volume (cm^3^)	Contralateral Side Volume, (cm^3^)	Median Difference (95% CI) (cm^3^)	*p*
1–3 months	103 (98.09%)	3.28 (1.33, 7.71)	3.43 (1.38, 5.92)	−0.15 (−0.98, 0.67)	0.301
3–6 months	96 (91.42%)	1.28 (0.76, 6.09)	2.67 (1.23, 6.24)	−1.39 (−3.02, 0.24)	0.169
6–12 months	87 (82.86%)	3.37 (2.04, 6.66)	4.37 (2.02, 6.28)	−1.00 (−2.15, 0.15)	0.092
>12 months	90 (85.71%)	4.07 (1.10, 5.04)	5.60 (1.02, 9.85)	−1.53 (−3.30, 0.24)	0.090

Data are presented as *n* (%) of the 105 included cases with available ultrasound measurements at each time window. The time windows are not mutually exclusive; the same patient may contribute data to more than one window. The denominator for all percentages is 105. Enrollment ended on 1 June 2025; the last follow-up was 1 July 2026.

## Data Availability

The data presented in this study are available on request from the corresponding author.
